# Attention-Deficit/Hyperactivity Disorder Medications and Long-Term Risk of Cardiovascular Diseases

**DOI:** 10.1001/jamapsychiatry.2023.4294

**Published:** 2023-11-22

**Authors:** Le Zhang, Lin Li, Pontus Andell, Miguel Garcia-Argibay, Patrick D. Quinn, Brian M. D’Onofrio, Isabell Brikell, Ralf Kuja-Halkola, Paul Lichtenstein, Kristina Johnell, Henrik Larsson, Zheng Chang

**Affiliations:** 1 Department of Medical Epidemiology and Biostatistics, Karolinska Institutet, Stockholm, Sweden; 2 Unit of Cardiology, Heart and Vascular Division, Department of Medicine, Karolinska University Hospital, Karolinska Institutet, Stockholm, Sweden; 3 School of Medical Sciences, Faculty of Medicine and Health, Örebro University, Örebro, Sweden; 4 Department of Applied Health Science, School of Public Health, Indiana University, Bloomington; 5 Department of Psychological and Brain Sciences, Indiana University, Bloomington

## Abstract

**Importance:**

Use of attention-deficit/hyperactivity disorder (ADHD) medications has increased substantially over the past decades. However, the potential risk of cardiovascular disease (CVD) associated with long-term ADHD medication use remains unclear.

**Objective:**

To assess the association between long-term use of ADHD medication and the risk of CVD.

**Design, Setting, and Participants:**

This case-control study included individuals in Sweden aged 6 to 64 years who received an incident diagnosis of ADHD or ADHD medication dispensation between January 1, 2007, and December 31, 2020. Data on ADHD and CVD diagnoses and ADHD medication dispensation were obtained from the Swedish National Inpatient Register and the Swedish Prescribed Drug Register, respectively. Cases included individuals with ADHD and an incident CVD diagnosis (ischemic heart diseases, cerebrovascular diseases, hypertension, heart failure, arrhythmias, thromboembolic disease, arterial disease, and other forms of heart disease). Incidence density sampling was used to match cases with up to 5 controls without CVD based on age, sex, and calendar time. Cases and controls had the same duration of follow-up.

**Exposure:**

Cumulative duration of ADHD medication use up to 14 years.

**Main Outcomes and Measures:**

The primary outcome was incident CVD. The association between CVD and cumulative duration of ADHD medication use was measured using adjusted odds ratios (AORs) with 95% CIs.

**Results:**

Of 278 027 individuals with ADHD aged 6 to 64 years, 10 388 with CVD were identified (median [IQR] age, 34.6 [20.0-45.7] years; 6154 males [59.2%]) and matched with 51 672 control participants without CVD (median [IQR] age, 34.6 [19.8-45.6] years; 30 601 males [59.2%]). Median (IQR) follow-up time in both groups was 4.1 (1.9-6.8) years. Longer cumulative duration of ADHD medication use was associated with an increased risk of CVD compared with nonuse (0 to ≤1 year: AOR, 0.99 [95% CI, 0.93-1.06]; 1 to ≤2 years: AOR, 1.09 [95% CI, 1.01-1.18]; 2 to ≤3 years: AOR, 1.15 [95% CI, 1.05-1.25]; 3 to ≤5 years: AOR, 1.27 [95% CI, 1.17-1.39]; and >5 years: AOR, 1.23 [95% CI, 1.12-1.36]). Longer cumulative ADHD medication use was associated with an increased risk of hypertension (eg, 3 to ≤5 years: AOR, 1.72 [95% CI, 1.51-1.97] and >5 years: AOR, 1.80 [95% CI, 1.55-2.08]) and arterial disease (eg, 3 to ≤5 years: AOR, 1.65 [95% CI, 1.11-2.45] and >5 years: AOR, 1.49 [95% CI, 0.96-2.32]). Across the 14-year follow-up, each 1-year increase of ADHD medication use was associated with a 4% increased risk of CVD (AOR, 1.04 [95% CI, 1.03-1.05]), with a larger increase in risk in the first 3 years of cumulative use (AOR, 1.08 [95% CI, 1.04-1.11]) and stable risk over the remaining follow-up. Similar patterns were observed in children and youth (aged <25 years) and adults (aged ≥25 years).

**Conclusions and Relevance:**

This case-control study found that long-term exposure to ADHD medications was associated with an increased risk of CVDs, especially hypertension and arterial disease. These findings highlight the importance of carefully weighing potential benefits and risks when making treatment decisions about long-term ADHD medication use. Clinicians should regularly and consistently monitor cardiovascular signs and symptoms throughout the course of treatment.

## Key Points

**Question:**

Is long-term use of attention-deficit/hyperactivity disorder (ADHD) medication associated with an increased risk of cardiovascular disease (CVD)?

**Findings:**

In this case-control study of 278 027 individuals in Sweden aged 6 to 64 years who had an incident ADHD diagnosis or ADHD medication dispensation, longer cumulative duration of ADHD medication use was associated with an increased risk of CVD, particularly hypertension and arterial disease, compared with nonuse.

**Meaning:**

Findings of this study suggest that long-term exposure to ADHD medications was associated with an increased risk of CVD; therefore, the potential risks and benefits of long-term ADHD medication use should be carefully weighed.

## Teaser

This nested case-control study examines the association between long-term use of medications to treat attention-deficit/hyperactivity disorder (ADHD) and the risk of cardiovascular disease.

## Introduction

Attention-deficit/hyperactivity disorder (ADHD) is a common psychiatric disorder characterized by developmentally inappropriate inattentiveness, impulsivity, and hyperactivity.^1,2^ Pharmacological therapy, including both stimulants and nonstimulants, is recommended as the first-line treatment for ADHD in many countries.^1,3^ The use of ADHD medication has increased greatly in both children and adults during the past decades.^4^ Although the effectiveness of ADHD medications has been demonstrated in randomized clinical trials (RCTs) and other studies,^5,6^ concerns remain regarding their potential cardiovascular safety.^7^ Meta-analyses of RCTs have reported increases in heart rate and blood pressure associated with both stimulant and nonstimulant ADHD medications.^5,7,8,9^

As RCTs typically evaluate short-term effects (average treatment duration of 75 days),^7^ it remains uncertain whether and to what extent the increases in blood pressure and heart rate associated with ADHD medication lead to clinically significant cardiovascular disease (CVD) over time. Longitudinal observational studies^10,11,12^ examining the association between ADHD medication use and serious cardiovascular outcomes have emerged in recent years, but the findings have been mixed. A meta-analysis^13^ of observational studies found no statistically significant association between ADHD medication and risk of CVD. However, the possibility of a modest risk increase cannot be ruled out due to several methodological limitations in these studies, including confounding by indication, immortal time bias, and prevalent user bias. Additionally, most of these studies had an average follow-up time of no more than 2 years.^13,14^ Thus, evidence regarding the long-term cardiovascular risk of ADHD medication use is still lacking.

Examining the long-term cardiovascular risk associated with ADHD medicine use is clinically important given that individuals with a diagnosis of ADHD, regardless of whether they receive treatment, face an elevated risk of CVD.^15^ Additionally, a substantial proportion of young individuals with ADHD continues to have impairing symptoms in adulthood,^16^ necessitating prolonged use of ADHD medication. Notably, studies have indicated a rising trend in the long-term use of ADHD medications, with approximately half of individuals using ADHD medication for over 5 years.^17^ Furthermore, evidence is lacking regarding how cardiovascular risk may vary based on factors such as type of CVD, type of ADHD medication, age, and sex.^13^ Therefore, there is a need for long-term follow-up studies to address these knowledge gaps and provide a more comprehensive understanding of the cardiovascular risks associated with ADHD medication use. This information is also crucial from a public health perspective, particularly due to the increasing number of individuals receiving ADHD medications worldwide.^4^

This study aimed to assess the association between cumulative use of ADHD medication up to 14 years and the risk of CVD by using nationwide health registers in Sweden. We hypothesized that longer cumulative use of ADHD medication would be associated with increased CVD risk. In addition, we aimed to examine whether the associations differ across types of ADHD medication, types of CVD, sex, and age groups.

## Methods

### Data Sources

We used data from several Swedish nationwide registers linked through unique personal identification numbers.^18^ Diagnoses were obtained from the National Inpatient Register,^19^ which contains data on inpatient diagnoses since 1973 and outpatient diagnoses since 2001. Information on prescribed medications was retrieved from the Swedish Prescribed Drug Register, which contains all dispensed medications in Sweden since July 2005 and includes information on drug identity based on the Anatomical Therapeutic Chemical (ATC) classification,^20^ dispensing dates, and free-text medication prescriptions. Socioeconomic factors were obtained from the Longitudinal Integrated Database for Health Insurance and Labour Market studies.^21^ Information on death was retrieved from the Swedish Cause of Death Register,^22^ which contains information on all deaths since 1952. The study was approved by the Swedish Ethical Review Authority. Informed patient consent is not required for register-based studies in Sweden. The study followed the Reporting of Studies Conducted Using Observational Routinely Collected Health Data–Pharmacoepidemiological Research (RECORD-PE) guideline.^23^

### Study Design

We conducted a nested case-control study of all individuals residing in Sweden aged 6 to 64 years who received an incident diagnosis of ADHD or ADHD medication dispensation^15^ between January 1, 2007, and December 31, 2020. The diagnosis of ADHD (*International Statistical Classification of Diseases and Related Health Problems, Tenth Revision* [*ICD-10*] code F90) was identified from the National Inpatient Register. Incident ADHD medication dispensation was identified from the Swedish Prescribed Drug Register and was defined as a dispensation after at least 18 months without any ADHD medication dispensation.^24^ Baseline (ie, cohort entry) was defined as the date of incident ADHD diagnosis or ADHD medication dispensation, whichever came first. Individuals with ADHD medication prescriptions for indications other than ADHD^25^ and individuals who emigrated, died, or had a history of CVD before baseline were excluded from the study. The cohort was followed until the case index date (ie, the date of CVD diagnosis), death, migration, or the study end date (December 31, 2020), whichever came first.

### Identification of Cases and Controls

**Figure 1.**
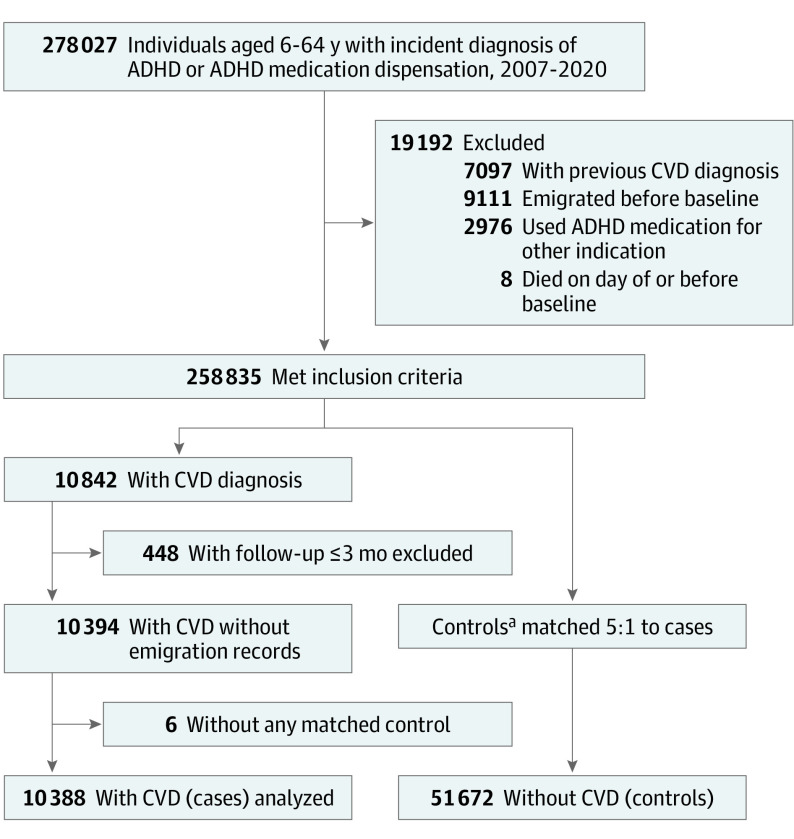
Selection of Cases and Matched Controls ADHD indicates attention-deficit/hyperactivity disorder; CVD, cardiovascular disease. ^a^Controls were derived from the same base cohort as the cases; thus, a case with a later date of CVD diagnosis could potentially serve as a control for another case in the study.

Within the study cohort, we identified cases as individuals with an incident diagnosis of any CVD (including ischemic heart diseases, cerebrovascular diseases, hypertension, heart failure, arrhythmias, thromboembolic disease, arterial disease, and other forms of heart disease; eTable 1 in Supplement 1) during follow-up. For each case, the date of their CVD diagnosis was assigned as the index date. Using incidence density sampling,^26^ up to 5 controls without CVD were randomly selected for each case from the base cohort of individuals with ADHD. The matching criteria included age, sex, and calendar time, ensuring that cases and controls had the same duration of follow-up from baseline to index date. Controls were eligible for inclusion if they were alive, living in Sweden, and free of CVD at the time when their matched case received a diagnosis of CVD, with the index date set as the date of CVD diagnosis of the matched case (Figure 1). Controls were derived from the same base cohort as the cases. Thus, a case with a later date of CVD diagnosis could potentially serve as a control for another case in the study.^26^

### Exposures

The main exposure was cumulative duration of ADHD medication use, which included all ADHD medications approved in Sweden during the study period, including stimulants (methylphenidate [ATC code N06BA04], amphetamine [ATC code N06BA01], dexamphetamine [ATC code N06BA02], and lisdexamfetamine [ATC code N06BA12]) as well as nonstimulants (atomoxetine [ATC code N06BA09] and guanfacine [ATC code C02AC02]). Duration of ADHD medication use was derived from a validated algorithm that estimates treatment duration from free text in prescription records.^25^ The cumulative duration of ADHD medication use was calculated by summing all days covered by ADHD medication between baseline and 3 months prior to the index date. The last 3 months before the index date were excluded to reduce reverse causation, as clinicians’ perception of potential cardiovascular risks may influence ADHD medication prescription. This time window was chosen because routine psychiatric practice in Sweden limits a prescription to a maximum 3 months at a time.^27^ Individuals with follow-up of less than 3 months were excluded.

### Statistical Analysis

We conducted conditional logistic regression analyses to estimate odds ratios (ORs) for the associations between cumulative durations of ADHD medication use and incident CVD. Crude ORs were adjusted for all matching variables (age, sex, and calendar time) by design. Adjusted ORs (AORs) were additionally controlled for country of birth (Sweden vs other), highest educational level (primary or lower secondary, upper secondary, postsecondary or postgraduate, or unknown; individuals aged <16 years were included as a separate category), and diagnoses of somatic (type 2 diabetes, obesity, dyslipidemia, and sleep disorders) and psychiatric comorbidities (anxiety disorders, autism spectrum disorder, bipolar disorder, conduct disorder, depressive disorder, eating disorders, intellectual disability, personality disorders, schizophrenia, and substance use disorders; eTable 1 in Supplement 1) before baseline. The association between cumulative ADHD medication use and incident CVD was assessed using both continuous and categorical measures (no ADHD medication use, 0 to ≤1, 1 to ≤2, 2 to ≤3, 3 to ≤5, and >5 years). To capture potential nonlinear associations, we used restricted cubic splines to examine ADHD medication use as a continuous measure throughout follow-up.^28^ The associations were examined in the full sample and stratified by age at baseline, that is, children or youth (<25 years old) and adults (≥25 years old). Furthermore, to evaluate the association with dosage of ADHD medication, we estimated the risk of CVD associated with each 1-year increase in use of ADHD medication across different dosage groups categorized by the average defined daily dose (DDD; for instance, 1 DDD of methylphenidate equals 30 mg) during follow-up.^29^

In subgroup analyses, we examined the associations between ADHD medication use and specific CVDs, including arrhythmias, arterial disease, cerebrovascular disease, heart failure, hypertension, ischemic heart disease, and thromboembolic disease (eTable 1 in Supplement 1). Additionally, we investigated the associations with CVD risk for the most commonly prescribed ADHD medications in Sweden, ie, methylphenidate, lisdexamfetamine, and atomoxetine, while adjusting for other ADHD medication use. We also examined sex-specific associations.

To further examine the robustness of our findings, we conducted 4 sensitivity analyses. First, we restricted the sample to ever users of ADHD medication to reduce unmeasured confounding between ADHD medication users and nonusers. Second, we assessed ADHD medication exposure over the entire follow-up period without excluding the 3 months prior to the index date. Third, to capture fatal cardiovascular events, we additionally included death by CVD in the outcome definition. Finally, we constructed a conditional logistic regression model that adjusted for propensity scores of ADHD medication use. Data management was performed using SAS, version 9.4 (SAS Institute Inc) and all analyses were performed using R, version 4.2.3 (R Foundation for Statistical Computing).

## Results

The study cohort consisted of 278 027 individuals with ADHD aged 6 to 64 years. The incidence rate of CVD was 7.34 per 1000 person-years. After applying exclusion criteria and matching, the analysis included 10 388 cases (median [IQR] age at baseline, 34.6 (20.0-45.7) years; 6154 males [59.2%] and 4234 females [40.8%]) and 51 672 matched controls (median [IQR] age at baseline, 34.6 [19.8-45.6] years; 30 601 males [59.2%] and 21 071 females [40.8%]) (Figure 1 and Table 1). Median (IQR) follow-up in both groups was 4.1 (1.9-6.8) years. Among the controls, 3363 had received a CVD diagnosis after their index dates. The most common types of CVD in cases were hypertension (4210 cases [40.5%]) and arrhythmias (1310 cases [12.6%]; eTable 2 in Supplement 1). Table 1 presents the sociodemographic information and somatic and psychiatric comorbidities in cases and controls. In general, cases had higher rates of somatic and psychiatric comorbidities and a lower level of educational attainment compared with controls.

**Table 1.** Characteristics of Cases and Matched Controls

Baseline characteristic	Full sample, No. (%)		Age <25 y, No. (%)		Age ≥25 y, No. (%)	
	Cases (n = 10 388)	Controls (n = 51 672)	Cases (n = 3406)	Controls (n = 17 027)	Cases (n = 6982)	Controls (n = 34 645)
Age, median (IQR), y	34.6 (20.0-45.7)	34.6 (19.8-45.6)	15.8 (12.3-19.6)	15.8 (12.3-19.6)	42.2 (34.4-49.3)	42.1 (34.4-49.1)
Follow-up, median (IQR), y	4.1 (1.9-6.8)	4.1 (1.9-6.8)	4.2 (1.9-7.0)	4.2 (1.9-6.9)	4.0 (1.9-6.8)	4.0 (1.9-6.8)
Sex						
Female	4234 (40.8)	21 071 (40.8)	1322 (38.8)	6607 (38.8)	2912 (41.7)	14 464 (41.7)
Male	6154 (59.2)	30 601 (59.2)	2084 (61.2)	10 420 (61.2)	4070 (58.3)	20 181 (58.3)
Country of birth						
Sweden	9520 (91.6)	46 805 (90.6)	3208 (94.2)	15 935 (93.6)	6312 (90.4)	30 870 (89.1)
Other	868 (8.4)	4867 (9.4)	198 (5.8)	1092 (6.4)	670 (9.6)	3775 (10.9)
Educational level						
Primary or lower secondary	3081 (29.7)	13 741 (26.6)	1182 (34.7)	5626 (33.0)	1899 (27.2)	8115 (23.4)
Upper secondary	4062 (39.1)	19 942 (38.6)	410 (12.0)	2313 (13.6)	3652 (52.3)	17 629 (50.9)
Postsecondary or postgraduate	1449 (13.9)	9064 (17.5)	58 (1.7)	367 (2.2)	1391 (19.9)	8697 (25.1)
People aged <16 y^a^	1680 (16.2)	8374 (16.2)	1680 (49.3)	8374 (49.2)	NA	NA
Unknown	116 (1.1)	551 (1.1)	76 (2.2)	347 (2.0)	40 (0.6)	204 (0.6)
Psychiatric comorbidities						
Anxiety disorders	3489 (33.6)	14 930 (28.9)	670 (19.7)	2811 (16.5)	2819 (40.4)	12 119 (35.0)
Autism spectrum disorder	803 (7.7)	4390 (8.5)	343 (10.1)	1853 (10.9)	460 (6.6)	2537 (7.3)
Bipolar disorder	830 (8.0)	3847 (7.4)	88 (2.6)	369 (2.2)	742 (10.6)	3478 (10.0)
Conduct disorder	189 (1.8)	799 (1.5)	139 (4.1)	634 (3.7)	50 (0.7)	165 (0.5)
Depressive disorder	3323 (32.0)	15 096 (29.2)	582 (17.1)	2574 (15.1)	2741 (39.3)	12 522 (36.1)
Eating disorders	363 (3.5)	1609 (3.1)	86 (2.5)	417 (2.4)	277 (4.0)	1192 (3.4)
Intellectual disability	276 (2.7)	1151 (2.2)	154 (4.5)	626 (3.7)	122 (1.7)	525 (1.5)
Personality disorders	1467 (14.1)	6137 (11.9)	167 (4.9)	640 (3.8)	1300 (18.6)	5497 (15.9)
Schizophrenia	516 (5.0)	2102 (4.1)	72 (2.1)	199 (1.2)	444 (6.4)	1903 (5.5)
Substance use disorders	3332 (32.1)	12 693 (24.6)	500 (14.7)	1638 (9.6)	2832 (40.6)	11 055 (31.9)
Somatic comorbidities						
Obesity	699 (6.7)	2186 (4.2)	217 (6.4)	563 (3.3)	482 (6.9)	1623 (4.7)
Type 2 diabetes	287 (2.8)	657 (1.3)	22 (0.6)	30 (0.2)	265 (3.8)	627 (1.8)
Dyslipidemia	81 (0.8)	404 (0.8)	6 (0.2)	12 (0.1)	75 (1.1)	392 (1.1)
Sleep disorders	642 (6.2)	2507 (4.9)	128 (3.8)	456 (2.7)	514 (7.4)	2051 (5.9)

Abbreviation: NA, not applicable.

^a^ The highest educational level is not relevant for individuals younger than 16 years.

**Figure 2.**
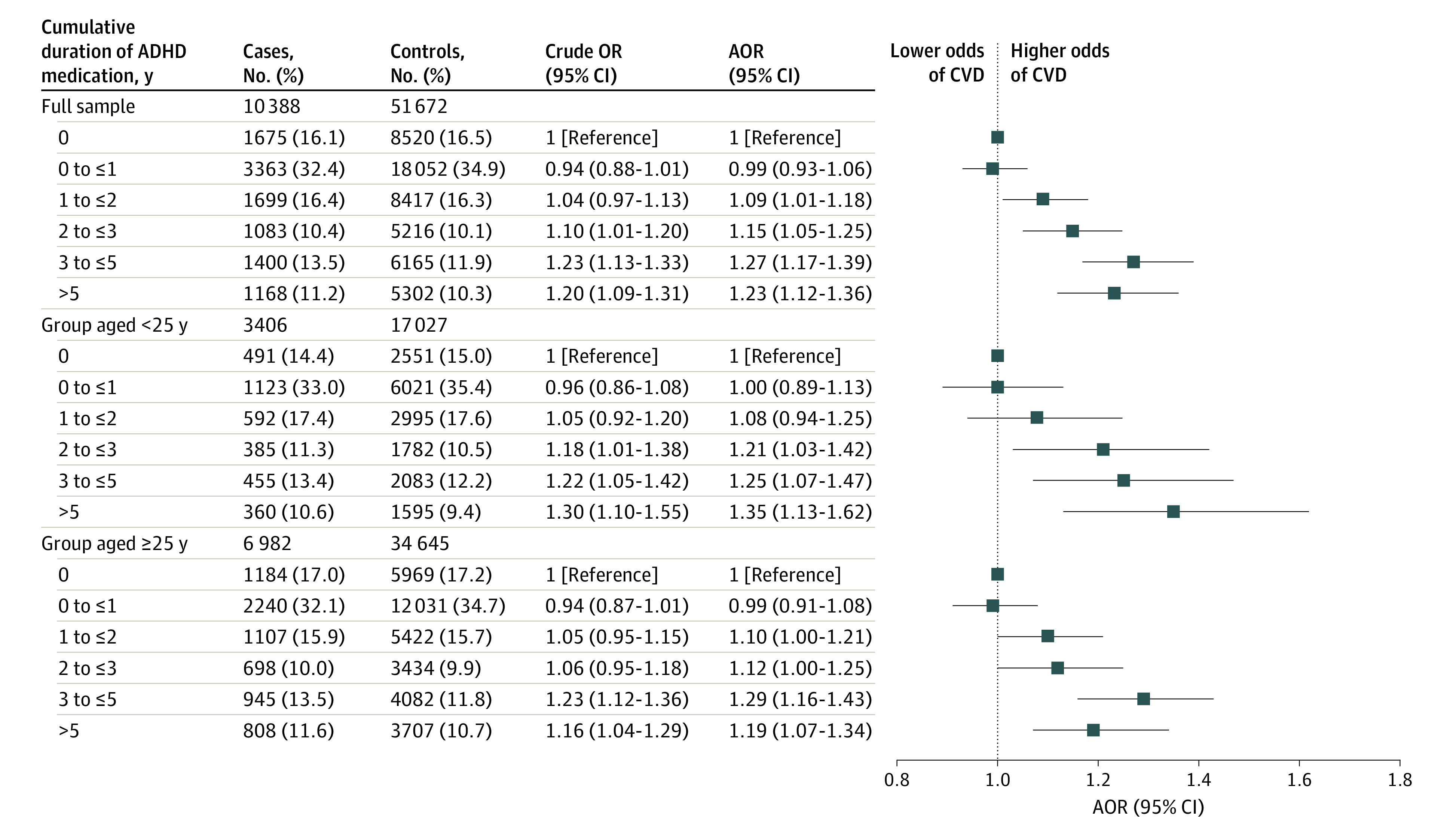
Risk of Cardiovascular Disease (CVD) Associated With Cumulative Duration of Attention-Deficit/Hyperactivity Disorder (ADHD) Medication Use Crude odds ratios (ORs) were based on cases and controls matched on age, sex, and calendar time. Adjusted ORs (AORs) were based on cases and controls matched on age, sex, and calendar time and adjusted for country of birth, educational level, somatic comorbidities (type 2 diabetes, obesity, dyslipidemia, and sleep disorders), and psychiatric comorbidities (anxiety disorders, autism spectrum disorder, bipolar disorder, conduct disorder, depressive disorder, eating disorders, intellectual disability, personality disorders, schizophrenia, and substance use disorders).

**Figure 3.**
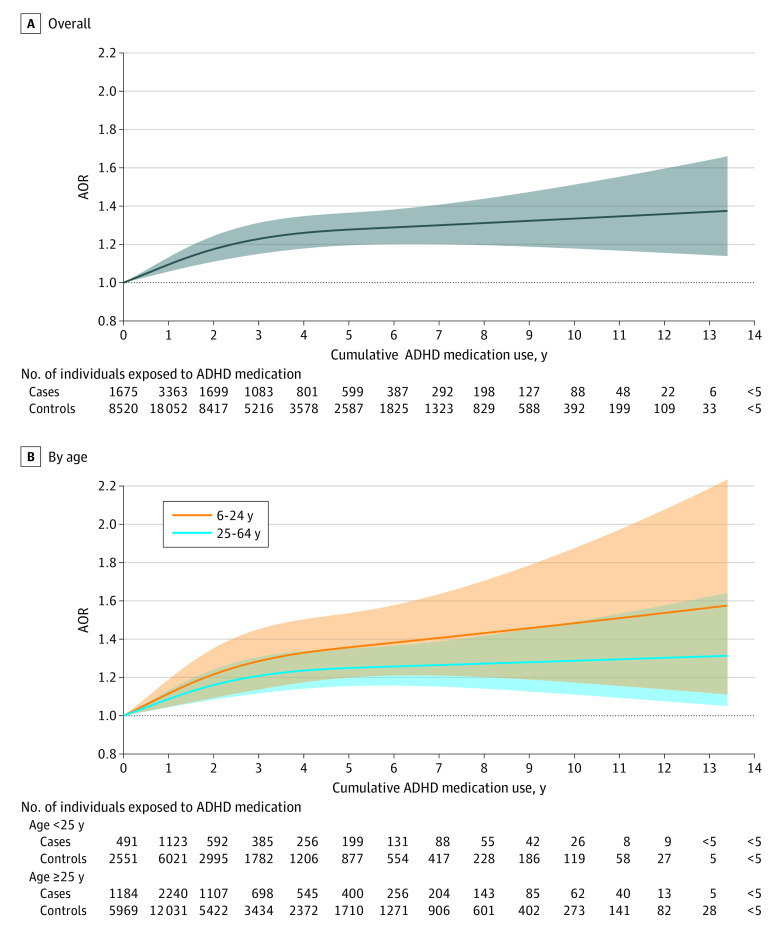
Association Between Cumulative Attention-Deficit/Hyperactivity Disorder Medication (ADHD) Use and Risk of Cardiovascular Disease The solid lines represent the adjusted odds ratios, and the shaded areas represent the 95% CIs. In restricted cubic splines analysis, knots were placed at the 10th, 50th, and 90th percentiles of ADHD medication use.

A similar proportion of cases (83.9%) and controls (83.5%) used ADHD medication during follow-up, with methylphenidate being the most commonly dispensed type, followed by atomoxetine and lisdexamfetamine. Longer cumulative duration of ADHD medication use was associated with an increased risk of CVD compared with nonuse (0 to ≤1 year: AOR, 0.99 [95% CI, 0.93-1.06]; 1 to ≤2 years: AOR, 1.09 [95% CI, 1.01-1.18]; 2 to ≤3 years: AOR, 1.15 [95% CI, 1.05-1.25]; 3 to ≤5 years: AOR, 1.27 [95% CI, 1.17-1.39]; and >5 years: AOR, 1.23 [95% CI, 1.12-1.36]) (Figure 2). The restricted cubic spline model suggested a nonlinear association, with the AORs increasing rapidly for the first 3 cumulative years of ADHD medication use and then becoming stable thereafter (Figure 3). Throughout the entire follow-up, each 1-year increase in the use of ADHD medication was associated with a 4% increased risk of CVD (AOR, 1.04 [95% CI, 1.03-1.05]), and the corresponding increase for the first 3 years was 8% (AOR, 1.08 [95% CI, 1.04-1.11]). We observed similar results when examining children or youth and adults separately (Figure 2). The restricted cubic spline model suggested a similar nonlinear association, with higher AORs in children or youth than in adults, but the 95% CIs largely overlapped (Figure 3). Furthermore, similar associations were observed for females and males (eFigure in Supplement 1). The dosage analysis showed that the risk of CVD associated with each 1 year of ADHD medication use increased with higher average DDDs. The risk was found to be statistically significant only among individuals with a mean dose of at least 1.5 times the DDD (eTable 3 in Supplement 1). For example, among individuals with a mean DDD of 1.5 to 2 or less (eg, for methylphenidate, 45 to ≤60 mg), each 1-year increase in ADHD medication use was associated with a 4% increased risk of CVD (AOR, 1.04 [95% CI, 1.02-1.05]). Among individuals with a mean DDD >2 (eg, for methylphenidate >60 mg), each 1-year increase in ADHD medication use was associated with 5% increased risk of CVD (AOR, 1.05 [95% CI, 1.03-1.06]).

When examining the risk for specific CVDs, we found that long-term use of ADHD medication (compared with no use) was associated with an increased risk of hypertension (AOR, 1.72 [95% CI, 1.51-1.97] for 3 to ≤5 years; AOR, 1.80 [95% CI 1.55-2.08] for >5 years) (Table 2), as well as arterial disease (AOR, 1.65 [95% CI, 1.11-2.45] for 3 to ≤5 years; AOR, 1.49 [95% CI 0.96-2.32] for >5 years). However, we did not observe any statistically significant increased risk for arrhythmias, heart failure, ischemic heart disease, thromboembolic disease, or cerebrovascular disease (Table 2). Furthermore, long-term use of methylphenidate (compared with no use) was associated with an increased risk of CVD (AOR, 1.20 [95% CI, 1.10-1.31] for 3 to ≤5 years; AOR, 1.19 [95% CI, 1.08-1.31]) for >5 years; eTable 4 in Supplement 1). Compared with no use, lisdexamfetamine was also associated with an elevated risk of CVD (AOR, 1.23 [95% CI, 1.05-1.44] for 2 to ≤3 years; AOR, 1.17 [95% CI, 0.98-1.40] for >3 years), while the AOR for atomoxetine use was significant only for the first year of use (1.07 [95% CI 1.01-1.13]; eTable 4 in Supplement 1).

**Table 2.** Risk of Cardiovascular Disease (CVD) Associated With Cumulative Duration of Attention-Deficit/Hyperactivity Disorder (ADHD) Medication Use, Stratified by Type of CVD Event

CVD event and cumulative duration of ADHD medication use, y	Cases, No. (%)^a^	Controls, No. (%)	Crude OR (95% CI)^b^	Adjusted OR (95% CI)^c^
Arrhythmias	1310	6499	NA	NA
0	233 (17.8)	1074 (16.5)	1 [Reference]	1 [Reference]
0 to ≤1	460 (35.1)	2396 (36.9)	0.89 (0.75-1.06)	0.91 (0.76-1.10)
1 to ≤2	236 (18.0)	1084 (16.7)	1.02 (0.83-1.26)	1.04 (0.84-1.28)
2 to ≤3	120 (9.2)	624 (9.6)	0.90 (0.70-1.16)	0.92 (0.70-1.19)
3 to ≤5	143 (10.9)	696 (10.7)	0.95 (0.75-1.22)	0.95 (0.74-1.23)
>5	118 (9.0)	625 (9.6)	0.85 (0.64-1.11)	0.87 (0.66-1.16)
Arterial disease	580	2890	NA	NA
0	66 (11.4)	450 (15.6)	1 [Reference]	1 [Reference]
0 to ≤1	203 (35.0)	1029 (35.6)	1.31 (0.98-1.77)	1.42 (1.04-1.93)
1 to ≤2	104 (17.9)	471 (16.3)	1.54 (1.10-2.17)	1.63 (1.15-2.32)
2 to ≤3	74 (12.8)	323 (11.2)	1.65 (1.13-2.40)	1.72 (1.17-2.53)
3 to ≤5	78 (13.4)	352 (12.2)	1.61 (1.10-2.36)	1.65 (1.11-2.45)
>5	55 (9.5)	265 (9.2)	1.44 (0.93-2.21)	1.49 (0.96-2.32)
Hypertension	4210	20 924	NA	NA
0	624 (14.8)	3478 (16.6)	1 [Reference]	1 [Reference]
0 to ≤1	1227 (29.1)	7123 (34.0)	0.94 (0.85-1.05)	1.06 (0.95-1.18)
1 to ≤2	699 (16.6)	3291 (15.7)	1.22 (1.08-1.38)	1.34 (1.18-1.52)
2 to ≤3	426 (10.1)	2132 (10.2)	1.19 (1.04-1.37)	1.30 (1.12-1.50)
3 to ≤5	650 (15.4)	2598 (12.4)	1.57 (1.38-1.79)	1.72 (1.51-1.97)
>5	584 (13.9)	2302 (11.0)	1.67 (1.45-1.92)	1.80 (1.55-2.08)
Cerebrovascular disease	705	3511	NA	NA
0	131 (18.6)	629 (17.9)	1 [Reference]	1 [Reference]
0 to ≤1	230 (32.6)	1152 (32.8)	0.96 (0.75-1.21)	0.93 (0.72-1.20)
1 to ≤2	121 (17.2)	590 (16.8)	0.99 (0.75-1.30)	0.97 (0.73-1.31)
2 to ≤3	58 (8.2)	314 (8.9)	0.89 (0.63-1.26)	0.85 (0.60-1.22)
3 to ≤5	101 (14.3)	432 (12.3)	1.12 (0.82-1.52)	1.10 (0.80-1.52)
>5	64 (9.1)	394 (11.2)	0.74 (0.52-1.05)	0.74 (0.51-1.07)
Heart failure	370	1833	NA	NA
0	64 (17.3)	299 (16.3)	1 [Reference]	1 [Reference]
0 to ≤1	120 (32.4)	584 (31.9)	0.95 (0.68-1.34)	1.04 (0.72-1.51)
1 to ≤2	41 (11.1)	298 (16.3)	0.63 (0.41-0.98)	0.68 (0.43-1.08)
2 to ≤3	47 (12.7)	192 (10.5)	1.14 (0.73-1.77)	1.22 (0.76-1.95)
3 to ≤5	49 (13.2)	248 (13.5)	0.93 (0.60-1.44)	1.05 (0.66-1.66)
>5	49 (13.2)	212 (11.6)	1.14 (0.72-1.81)	1.23 (0.76-1.99)
Ischemic heart disease	493	2437	NA	NA
0	83 (16.8)	380 (15.6)	1 [Reference]	1 [Reference]
0 to ≤1	164 (33.3)	838 (34.4)	0.89 (0.67-1.19)	0.94 (0.69-1.28)
1 to ≤2	60 (12.2)	384 (15.8)	0.70 (0.48-1.02)	0.72 (0.49-1.06)
2 to ≤3	55 (11.2)	256 (10.5)	1.00 (0.67-1.47)	1.09 (0.73-1.65)
3 to ≤5	68 (13.8)	285 (11.7)	1.14 (0.78-1.64)	1.20 (0.82-1.77)
>5	63 (12.8)	294 (12.1)	0.98 (0.66-1.45)	1.06 (0.70-1.60)
Thromboembolic disease	1254	6237	NA	NA
0	216 (17.2)	1048 (16.8)	1 [Reference]	1 [Reference]
0 to ≤1	428 (34.1)	2150 (34.5)	0.98 (0.82-1.17)	0.97 (0.80-1.18)
1 to ≤2	202 (16.1)	1057 (16.9)	0.94 (0.76-1.17)	0.93 (0.74-1.17)
2 to ≤3	131 (10.4)	624 (10.0)	1.03 (0.81-1.32)	1.07 (0.82-1.38)
3 to ≤5	163 (13.0)	731 (11.7)	1.11 (0.87-1.41)	1.08 (0.84-1.39)
>5	114 (9.1)	627 (10.1)	0.88 (0.67-1.15)	0.84 (0.63-1.12)

Abbreviation: OR, odds ratio.

^a^ Multiple incident diagnoses can be assigned simultaneously on the same day as incident CVD. As a result, the percentages of individual diagnoses may add up to more than 100%.

^b^ Crude ORs are based on cases and controls matched on age, sex, and calendar time.

^c^ Adjusted ORs are based on cases and controls matched on age, sex, and calendar time and adjusted for country of birth, highest educational level, somatic comorbidities (type 2 diabetes, obesity, dyslipidemia, and sleep disorders), and psychiatric comorbidities (anxiety disorders, autism spectrum disorder, bipolar disorder, conduct disorder, depressive disorder, eating disorders, intellectual disability, personality disorders, schizophrenia, and substance use disorders).

In sensitivity analyses, we observed a similar pattern of estimates when the analysis was restricted to ever users of ADHD medications. Significantly increased risk of CVD was found when comparing ADHD medication use for 1 year or less with use for 3 to 5 or less years (AOR, 1.28 (95% CI, 1.18-1.38) or for use for more than 5 years (AOR, 1.24 [95% CI, 1.13-1.36]) (eTable 5 in Supplement 1). When assessing ADHD medication use across the entire follow-up period, and compared with no use, the pattern of estimates was similar to the main analysis (3 to ≤5 years: AOR, 1.28 [95% CI, 1.18-1.39]; >5 years: AOR, 1.25 [95% CI, 1.14-1.37]) (eTable 5 in Supplement 1). The analysis that included cardiovascular death as a combined outcome also had results similar to the main analysis. Moreover, when adjusting for propensity scores of ADHD medication use, the findings remained consistent (eTable 5 in Supplement 1).

## Discussion

This large, nested case-control study found an increased risk of incident CVD associated with long-term ADHD medication use, and the risk increased with increasing duration of ADHD medication use. This association was statistically significant both for children and youth and for adults, as well as for females and males. The primary contributors to the association between long-term ADHD medication use and CVD risk was an increased risk of hypertension and arterial disease. Increased risk was also associated with stimulant medication use.

We found individuals with long-term ADHD medication use had an increased risk of incident CVD in a dose-response manner in the first 3 years of cumulative ADHD medication use. To our knowledge, few previous studies have investigated the association between long-term ADHD medication use and the risk of CVD with follow-up of more than 2 years.^13^ The only 2 prior studies with long-term follow-up (median, 9.5 and 7.9 years^30,31^) found an average 2-fold and 3-fold increased risk of CVD with ADHD medication use compared with nonuse during the study period, yet 1 of the studies^30^ included only children, and participants in the other study^31^ were not the general population of individuals with ADHD (including those with ADHD and long QT syndrome). Furthermore, both studies were subject to prevalent user bias. Results from the current study suggest that the CVD risk associated with ADHD medication use (23% increased risk for >5 years of ADHD medication use compared with nonuse) is lower than previously reported.^30,31^ Furthermore, we observed that the increased risk stabilized after the first several years of medication use and persisted throughout the 14-year follow-up period.

The association between ADHD medication use and CVD was significant for hypertension and arterial disease, while no significant association was observed with other types of cardiovascular events. To our knowledge, only 1 previous study^12^ has examined the association between ADHD medication use and clinically diagnosed hypertension, and it found an increased risk, although the increase was not statistically significant. Furthermore, increased blood pressure associated with ADHD medication use has been well documented.^7,9^ One study^32^ found that blood pressure was mainly elevated during the daytime, suggesting that the cardiovascular system may recover at night. However, the cross-sectional nature of that study cannot preclude a long-term risk of clinically diagnosed hypertension associated with ADHD medication use. We also identified an increased risk for arterial disease. To date, no previous study has explored the association between ADHD medication use and arterial disease. A few studies have reported that ADHD medication may be associated with changes in serum lipid profiles, but the results were not consistent.^33,34^ Further research is needed on the potential implications of ADHD medications for individuals’ lipid profiles. We did not observe any association between ADHD medication use and the risk of arrhythmias. A recent systematic review of observational studies of ADHD medication use reported an elevated risk of arrhythmias, but the finding was not statistically significant.^13^ A review of RCTs also found that the use of stimulants was associated with an average increase in heart rate of 5.7 beats/min,^9^ but no evidence of prolonged QT interval or tachycardia was found based on electrocardiograms.^7^ Additionally, it is worth noting that some individuals receiving ADHD medications might be prescribed antiarrhythmic β-blockers to alleviate palpitation symptoms, thus potentially attenuating an association between ADHD medications and arrhythmias. Nevertheless, the absence of an association between ADHD medication use and clinically diagnosed arrhythmias in the present study does not rule out an increased risk for mild arrhythmias or subclinical symptoms, as palpitations and sinus tachycardia are not routinely coded as arrhythmia diagnoses. Further research is necessary to replicate our findings.

Regarding types of ADHD medication, findings of the present study suggest that increasing cumulative durations of methylphenidate and lisdexamfetamine use were associated with incident CVD, while the associations for atomoxetine were statistically significant only for the first year of use. Previous RCTs have reported increased blood pressure and heart rate with methylphenidate, lisdexamfetamine, and atomoxetine,^5,35,36^ but the mechanisms behind these adverse effects are still a topic of debate; there might be differences in cardiovascular adverse effects in stimulants vs nonstimulants.^37^

We found that the association between cumulative duration of ADHD medication use and CVD was similar in females and males. Previous investigations exploring sex-specific association found higher point estimates in females, although the differences were not statistically significant.^13^ Research has indicated that females diagnosed with ADHD may demonstrate different comorbidity patterns and potentially have different responses to stimulant medications compared with males.^38,39,40^ Therefore, additional studies are needed to explore and better understand the potential sex-specific differences in cardiovascular responses to ADHD medications.

### Strengths and Limitations

A strength of this study is that data on ADHD medication prescriptions and CVD diagnoses were recorded prospectively, so the results were not affected by recall bias. The findings should, however, be interpreted in the context of several limitations. First, our approach for identification of patients with CVD was based on recorded diagnoses and there could be under ascertainment of cardiovascular diagnoses in the registers used. This means that some controls may have had undiagnosed CVD that did not yet require medical care, which would tend to underestimate associations between ADHD medication use and CVD. Second, exposure misclassification may have occurred if patients did not take their medication as prescribed. This misclassification, if nondifferential, would tend to reduce ORs such that the estimates we observed were conservative. Third, while we accounted for a wide range of potential confounding variables, considering the observational nature of the study and the possibility of residual confounding, we could not prove causality. It is possible that the association observed might have been affected by time-varying confounders. For example, other psychotropic medications and lifestyle factors could have affected both ADHD medication use and the occurrence of cardiovascular events.^41,42^ Confounding by ADHD severity is also a potential factor to consider, as individuals with more severe ADHD symptoms may have more comorbidities and a less healthy lifestyle, which could affect the risk of CVD. Fourth, the study did not examine the risk of CVD among individuals with preexisting CVD. Individuals with preexisting CVD represent a distinct clinical group that requires careful monitoring; thus, evaluating the risk among them necessitates a different study design that carefully considers the potential impact of prior knowledge and periodic monitoring. Finally, the results by type of ADHD medication and type of CVD need to be replicated by studies with larger sample sizes.

## Conclusions

The results of this population-based case-control study with a longitudinal follow-up of 14 years suggested that long-term use of ADHD medication was associated with an increased risk of CVD, especially hypertension and arterial disease, and the risk was higher for stimulant medications. These findings highlight the importance of carefully weighing potential benefits and risks when making treatment decisions on long-term ADHD medication use. Clinicians should be vigilant in monitoring patients, particularly among those receiving higher doses, and consistently assess signs and symptoms of CVD throughout the course of treatment. Monitoring becomes even more crucial considering the increasing number of individuals engaging in long-term use of ADHD medication.

## Notes

Supplement 1. **eTable 1.** International Classification of Diseases (ICD) Codes from the Swedish National Inpatient Register **eTable 2.** Type of Cardiovascular Disease in Cases **eTable 3.** Risk of CVD Associated With ADHD Medication Use Across Different Average Defined Daily Doses **eTable 4.** Risk of CVD Associated With Cumulative Duration of Use of Different Types of ADHD Medications **eTable 5.** Sensitivity Analyses of CVD Risk Associated With Cumulative Use of ADHD Medications, Based On Different Cohort, Exposure, and Outcome Definitions **eFigure.** Risk of CVD Associated With Cumulative Use of ADHD Medications, Stratified by Sex

Supplement 2.
Data Sharing Statement
