# Supplementary material for: Attention-Deficit/Hyperactivity Disorder Medications and Long-Term Risk of Cardiovascular Diseases
Source: JAMA Psychiatry. 2023 Nov 22;81(2):178–87. doi: 10.1001/jamapsychiatry.2023.4294 (PMC10851097; doi:10.1001/jamapsychiatry.2023.4294)
Supplement: Supplement 1. — eTable 1. International Classification of Diseases (ICD) Codes from the Swedish National Inpatient Register eTable 2. Type of Cardiovascular Disease in Cases eTable 3. Risk of CVD Associated With ADHD Medication Use Across Different Average Defined Daily Doses eTable 4. Risk of CVD Associated With Cumulative Duration of Use of Different Types of ADHD Medications eTable 5. Sensitivity Analyses of CVD Risk Associated With Cumulative Use of ADHD Medications, Based On Different Cohort, Exposure, and Outcome Definitions eFigure. Risk of CVD Associated With Cumulative Use of ADHD Medications, Stratified by Sex [file jamapsychiatry-e234294-s001.pdf]

Supplemental Online Content

Zhang L, Li L, Andell P, et al. Medications for attention-deficit/hyperactivity disorder and long-term risk of cardiovascular diseases. *JAMA Psychiatr*. Published online November 22, 2023. doi:10.1001/jamapsychiatry.2023.4294

eTable 1. *International Classification of Diseases* (*ICD*) Codes from the Swedish National Patient Register

eTable 2. Type of Cardiovascular Disease in Cases

eTable 3. Risk of CVD Associated With ADHD Medication Use Across Different Defined Daily Doses

eTable 4. Risk of CVD Associated With Cumulative Duration of Use of Different Types of ADHD Medications

eTable 5. Sensitivity Analyses of CVD Risk Associated With Cumulative Use of ADHD Medications, Based On Different Cohort, Exposure, and Outcome Definitions

eFigure. Risk of CVD Associated With Cumulative Use of ADHD Medications, Stratified by Sex

This supplemental material has been provided by the authors to give readers additional information about their work.

© 2023 Zhang L et al. *JAMA Psychiatry*.

eTable 1. ***International Classification of Diseases*** **(*ICD*) Codes from the Swedish National Patient Register**

	ICD-8 Codes	ICD-9 Codes	ICD-10 Codes
Any CVD	39-43, 440-445, 450-453, 458	39-43, 440-445, 451	I0-I3, I42, I44.1, I44.2, I46, I47.0-I47.2, I48, I49.0, I49.5, I49.8, I50, I6, I70-I75, I80
Ischemic heart disease	410-414	410-414	I20-I24, I25.1, I25.2, I25.5-I25.9
Cerebrovascular disease	430-434, 436-438	430-434, 436-438	I6
Hypertension	400-404	401-405	I1
Heart failure	428	428	I50, I42
Arrhythmias	-	426A/B, 427A/B/D/E/F/W	I44.1, I44.2, I46, I47.0-I47.2, I48, I49.0, I49.5, I49.8
Thromboembolic disease	450, 451	415B, 451B	I26, I80
Arterial disease	440-444	440-444	I70-I74
Somatic comorbidities			
Obesity	277	278.A, 278.B	E65, E66
Type 2 diabetes mellitus	250	250	E11
Hyperlipidemia	279	272	E78
Sleep problems	347, 780.60	347,780F	F51, G47.0- G47.4, G47.8, G47.9
Psychiatric comorbidities			
Anxiety disorder	300.0	300.00,300.02	F40-F41
Autism spectrum disorder	-	299	F84
Bipolar disorder	296.1, 296.3, 296.8	296A/ C/D/E/W	F30-F31
Conduct disorder	-	312	F91
Depressive disorder	296.2, 298.0,300.4	296B,300E	F32-33
Eating disorders	306.5x	307.5	F50
Intellectual disability	310-315	317-319	F7
Personality disability	301	301	F60-62, F69
Schizophrenia	295 except 295.7	295A-E/G/W/X	F2
Substance use disorder	291, 303, 304	291, 292, 304, 305A, 305X	F10-16, F18-F19

eTable 2. Type of Cardiovascular Disease in Cases

Type of cardiovascular disease	Case (n=10 388) [a]
Arrhythmias	1 310 (12.6%)
Arterial disease	580 (5.6%)
Cerebrovascular diseases	705 (6.8%)
Heart failure	370 (3.6%)
Hypertension	4 210 (40.5%)
Ischemic heart disease	493 (4.7%)
Thromboembolic disease	1 254 (12.1%)
Other	1 820 (17.5%)

[a] Multiple diagnoses can be given simultaneously, which means that the percentages of individual diagnoses may add up to more than 100%.

eTable 3. Risk of CVD Associated With ADHD Medication Use Across Different Defined Daily Doses

Average DDD [a]	Case<br>n=10 388	Control<br>n=51 672	Adjusted ORs<br>(95% CI) [b, c]
0	1679 (16.2%)	8559 (16.6%)	n/a
0<DDD≤1.0	2459 (23.7%)	14243 (27.6%)	1.00 (0.97-1.03)
1.0<DDD≤1.5	1819 (17.5%)	9811 (19.0%)	1.01 (0.99-1.03)
1.5< DDD≤2.0	1482 (14.3%)	7156 (13.8%)	1.04 (1.02-1.05)
>2.0	2949 (28.4%)	11903 (23.0%)	1.05 (1.03-1.06)

Abbreviations: ADHD, attention-deficit/hyperactivity disorder; CI, confidence interval; CVD, cardiovascular disease; DDD, defined daily dose.

[a] Average DDD was defined as the mean DDD per day during exposed time periods. For example, 1 DDD of methylphenidate equals 30 mg and 1 DDD of atomoxetine equals 80 mg.

[b] The OR presents the odds ratio of CVD associated with one-year increase in ADHD medication use among individuals with different average dosages. For instance, among individuals with average DDD>2.0, each one-year increase in ADHD medication use was associated with 5% increased risk of CVD (OR=1.05, 95% CI 1.03-1.06).

[c] Adjusted ORs were based on cases and controls matched on age, sex, and calendar time and adjusted for country of birth, highest educational level, somatic comorbidities, including type 2 diabetes mellitus, obesity, dyslipidemia, sleep disorders, psychiatric comorbidities, including anxiety disorders, autism spectrum disorder, bipolar disorder, conduct disorder, depressive disorder, eating disorders, intellectual disability, personality disorders, schizophrenia, and substance use disorders.

eTable 4. Risk of CVD Associated With Cumulative Duration of Use of Different Types of ADHD Medications

Years of ADHD medication use	Case (n=10 388)	Control (n=51 672)	Adjusted ORs (95% CI)[a]
Methylphenidate			
0	2 468 (23.8%)	12 369 (23.9%)	1
0<duration ≤1	3 563 (34.3%)	18 931 (36.6%)	0.98 (0.92-1.04)
1<duration ≤2	1 488 (14.3%)	7 240 (14.0%)	1.08 (1.00-1.16)
2<duration ≤3	885 (8.5%)	4 253 (8.2%)	1.10 (1.01-1.20)
3<duration ≤5	1 102 (10.6%)	4 913 (9.5%)	1.20 (1.10-1.31)
>5	882 (8.5%)	3 966 (7.7%)	1.19 (1.08-1.31)
Atomoxetine [b]			
0	7 877 (75.8%)	39 999 (77.4%)	1
0<duration ≤1	1 968 (18.9%)	9 124 (17.7%)	1.07 (1.01-1.13)
1<duration ≤2	271 (2.6%)	1 242 (2.4%)	1.09 (0.95-1.25)
2<duration ≤3	113 (1.1%)	545 (1.1%)	1.09 (0.88-1.34)
>3	159 (1.5%)	762 (1.5%)	1.08 (0.90-1.29)
Lisdexamphetamine [b]			
0	8 354 (80.4%)	42 515 (82.3%)	1
0<duration ≤1	1 270 (12.2%)	5 769 (11.2%)	1.13 (1.05-1.21)
1<duration ≤2	384 (3.7%)	1 768 (3.4%)	1.11 (0.99-1.25)
2<duration ≤3	213 (2.1%)	888 (1.7%)	1.23 (1.05-1.44)
>3	167 (1.6%)	732 (1.4%)	1.17 (0.98-1.40)

Abbreviations: ADHD, attention-deficit/hyperactivity disorder; CI, confidence interval; CVD, cardiovascular disease
[a] Adjusted ORs are based on cases and controls matched on age, sex, and calendar time and adjusted for country of birth, highest educational level, somatic comorbidities, including type 2 diabetes mellitus, obesity, dyslipidemia, sleep disorders, psychiatric comorbidities, including anxiety disorders, autism spectrum disorder, bipolar disorder, conduct disorder, depressive disorder, eating disorders, intellectual disability, personality disorders, schizophrenia, and substance use disorders, and other ADHD medication use.
[b] The exposure category of 3<duration ≤5 years and duration>5 years were combined for atomoxetine and lisdexamphetamine due to a limited number of individuals in these categories. Lisdexamphetamine was not used in Sweden until 2013.

eTable 5. Sensitivity Analyses of CVD Risk Associated With Cumulative Duration of ADHD Medication Use, Using Different Cohort, Exposure, and Outcome Definitions

Cumulative duration in years	Case	Control	Adjusted ORs (95% CI) [a]
Ever users	n=8 713	n=43 152	
0<duration ≤1	3 363 (38.6%)	18 052 (41.8%)	1
1<duration ≤2	1 699 (19.5%)	8 417 (19.5%)	1.09 (1.01-1.16)
2<duration ≤3	1 083 (12.4%)	5 216 (12.1%)	1.14 (1.05-1.24)
3<duration ≤5	1 400 (16.1%)	6 165 (14.3%)	1.28 (1.18-1.38)
>5	1 168 (13.4%)	5 302 (12.3%)	1.24 (1.13-1.36)
Exposure window defined as entire follow-up [b]	n=10 832	n=53 887	
0	1766 (16.3%)	8944 (16.6%)	1
0<duration ≤1	3498 (32.3%)	18700 (34.7%)	1.00 (0.94-1.07)
1<duration ≤2	1738 (16.0%)	8631 (16.0%)	1.10 (1.02-1.19)
2<duration ≤3	1117 (10.3%)	5437 (10.1%)	1.14 (1.05-1.25)
3<duration ≤5	1449 (13.4%)	6443 (12.0%)	1.28 (1.18-1.39)
>5	1264 (11.7%)	5732 (10.6%)	1.25 (1.14-1.37)
Combined outcome of CVD diagnosis and death	n=10 615	n=51 672	
0	1720 (16.2%)	8791 (16.7%)	1
0<duration ≤1	3434 (32.4%)	18449 (34.9%)	0.99 (0.93-1.06)
1<duration ≤2	1738 (16.4%)	8427 (16.0%)	1.11 (1.03-1.21)
2<duration ≤3	1103 (10.4%)	5410 (10.2%)	1.13 (1.03-1.23)
3<duration ≤5	1425 (13.4%)	6253 (11.8%)	1.28 (1.18-1.40)
>5	1195 (11.3%)	5464 (10.3%)	1.22 (1.11-1.34)
Propensity score-adjusted analysis	n=10 388	n=51 672	
0	1675 (16.1%)	8520 (16.5%)	1
0<duration ≤1	3363 (32.4%)	18052 (34.9%)	0.97 (0.91-1.04)
1<duration ≤2	1699 (16.4%)	8417 (16.3%)	1.08 (1.00-1.17)
2<duration ≤3	1083 (10.4%)	5216 (10.1%)	1.13 (1.04-1.24)
3<duration ≤5	1400 (13.5%)	6165 (11.9%)	1.27 (1.17-1.38)
>5	1168 (11.2%)	5302 (10.3%)	1.24 (1.13-1.36)

Abbreviations: ADHD, attention-deficit/hyperactivity disorder; CI, confidence interval; CVD, cardiovascular disease; DDD, defined daily dose.

[a] Adjusted ORs were based on cases and controls matched on age, sex, and calendar time and adjusted for country of birth, highest educational level, somatic comorbidities, including type 2 diabetes mellitus, obesity, dyslipidemia, sleep disorders, psychiatric comorbidities, including anxiety disorders, autism spectrum disorder, bipolar disorder, conduct disorder, depressive disorder, eating disorders, intellectual disability, personality disorders, schizophrenia, and substance use disorders.

[b] The study population included individuals with follow-up less than 3 months.

eFigure. Risk of CVD Associated With Cumulative Duration of ADHD Medication Use, Stratified by Sex

Cumulative duration in years	Case	Control	Adjusted ORs (95% CI) [a]
Female	n=4 234	n=21 071	
0	659 (15.6%)	3 477 (16.5%)	1
0<duration ≤1	1 390 (32.8%)	7 447 (35.3%)	1.05 (0.95-1.17)
1<duration ≤2	704 (16.6%)	3 411 (16.2%)	1.17 (1.03-1.32)
2<duration ≤3	417 (9.8%)	2 104 (10.0%)	1.14 (0.99-1.32)
3<duration ≤5	600 (14.2%)	2 497 (11.9%)	1.43 (1.25-1.63)
>5	464 (11.0%)	2 135 (10.1%)	1.28 (1.10-1.49)
Male	n=6 154	n=30 601	
0	1 016 (16.5%)	5 043 (16.5%)	1
0<duration ≤1	1 973 (32.1%)	10 605 (34.7%)	0.95 (0.87-1.04)
1<duration ≤2	995 (16.2%)	5 006 (16.4%)	1.04 (0.94-1.15)
2<duration ≤3	666 (10.8%)	3 112 (10.2%)	1.14 (1.02-1.29)
3<duration ≤5	800 (13.0%)	3 668 (12.0%)	1.18 (1.05-1.32)
>5	704 (11.4%)	3 167 (10.3%)	1.20 (1.06-1.36)

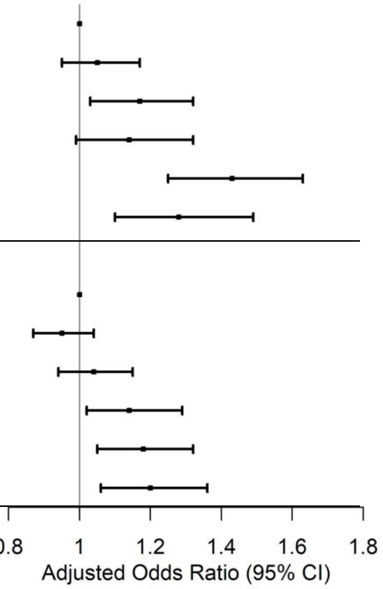

Abbreviations: ADHD, attention-deficit/hyperactivity disorder; CI, confidence interval; CVD, cardiovascular disease
[a] Adjusted ORs were based on cases and controls matched on age, sex, and calendar time and adjusted for country of birth, highest educational level, somatic comorbidities, including type 2 diabetes mellitus, obesity, dyslipidemia, sleep disorders, psychiatric comorbidities, including anxiety disorders, autism spectrum disorder, bipolar disorder, conduct disorder, depressive disorder, eating disorders, intellectual disability, personality disorders, schizophrenia, and substance use disorders.
