# Supplementary material for: Attention-Deficit/Hyperactivity Disorder Medications and Long-Term Risk of Cardiovascular Diseases
Source: JAMA Psychiatry. 2023 Nov 22;81(2):178–87. doi: 10.1001/jamapsychiatry.2023.4294 (PMC10851097; doi:10.1001/jamapsychiatry.2023.4294)
Supplement: Supplement 2. — Data Sharing Statement [file jamapsychiatry-e234294-s002.pdf]

Data Sharing Statement

Zhang. ADHD Medications and Long-Term Risk of Cardiovascular Diseases. *JAMA Psychiatry*. Published November 22, 2023. doi:10.1001/jamapsychiatry.2023.4294

Data
Data available: No

Additional Information
Explanation for why data not available: The data supporting the findings of this study are available from Statistics Sweden and The Swedish National Board of Health and Welfare; however, due to ethical permissions and restrictions, these data are not publicly available.
